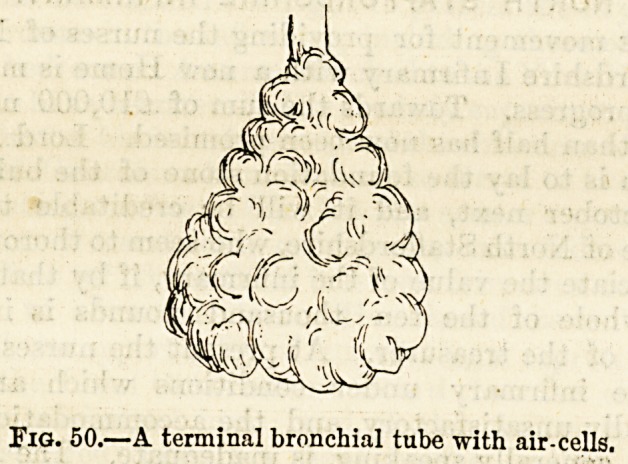# The Hospital. Nursing Section

**Published:** 1902-07-12

**Authors:** 


### The Hospital.

### Nursing Section.

Contributions for this Section of "The Hospital" should be addressed to the Editor, "The Hospital" Nursing Section, 28 & 29 Southampton Street, Strand, London, W.C.

No. 824.—Vol. XXXII. *SATURDAY, JULY 12, 1902.*

### Notes on News from the Nursing World.

### THE KING AND HIS NURSES.

The Prince of Wales, in the course of his eloquent and feeling speech at the opening of the Henriette Raphael Nurses' Home, at Guy's Hospital, on Monday afternoon, testified in a striking manner to the capacity and devotion shown by the nurse of the present day. First of all, his Royal Highness said, "We who have watched at the sick bed of the King fully realise how much, humanly speaking, is due to the patient and highly trained nursing which it has been his Majesty's good fortune to enjoy." The Prince, proceeding to express his satisfaction that the rôle of the nurse in the sick-room has been fully recognised, asked if there were not many "who like himself would throughout their lives remember with the deepest gratitude the soothing comfort, indeed, the blessing of efficient nursing." This is a graceful compliment to the Sister from St. Mary's Hospital who on more than one occasion has nursed the Heir Apparent. Miss Fletcher, who shares the duties of the Royal sick-room with Miss Haines, is a native of Abram, near Wigan.

### THE QUEEN AND THE PENSION FUND NURSE.

It is well known that the Queen is not content to be an ornamental President of the Royal National Pension Fund for Nurses. Ever since the time when as Princess of Wales she graciously consented to become the head of the organisation, she has manifested the keenest interest in all its movements. How thoroughly she desires that the members of the Fund should feel that she is in touch with them is shown by an incident which has come under our notice. Last week a nurse at Redcar, who is a member of the Fund, telegraphed her congratulations on the King's improvement to the Queen as President. To her intense delight she received on the following day, from Buckingham Palace, the following telegram, "The Queen thanks you for kind sympathy. The King is progressing most favourably."

### THE WOMEN'S MEMORIAL TO QUEEN VICTORIA.

The amount subscribed to the Women's Memorial to Queen Victoria on behalf of the Queen's Nurses has reached £48,000. This sum represents the contributions of upwards of two and a half million people. We hope to see it substantially augmented; but the popularity of the movement has been conclusively demonstrated both by the number of the subscribers, and also by the aggregate amount collected. Recent contributions include £487 8s. 3d. from Lady Llangattock as president for Monmouthshire, and £716 5s. 3d. from Mrs. Boulnois as mayoress of St. Marylebone.

### VICTORIAN ORDER OF NURSES FOR CANADA.

The High Commissioner for Canada sends us a copy of the report of the Board of Governors of the Victorian Order of Nurses for the Dominion for 1901, from which we learn that owing to the great increase in the amount of work devolving upon the chief lady superintendent, the executive have recommended that an assistant superintendent be appointed, whose chief duty it will be to give furlough to the nurses and to inspect the nursing in hospitals. This means, of course, that the duties discharged by the Order of Nurses in Canada continues to be more widely appreciated. The report says: "To the district superintendents, head nurses, and nurses the board feels deeply indebted for the earnest and conscientious spirit in which they have carried on the work for which the Order exists. To numberless homes they have brought help, comfort, and encouragement as well as most useful instruction in the proper care of the sick in their own homes." New branches have been started at Pictou, N.S., and at Dauphin, N.W.T. In Pictou, a wing of the Marine Hospital is now supplied with nursing service by the Victorian Order, whereas before no nursing was available. During the year the chief lady superintendent made 28 inspections, 11 places were visited with a view to organising, three and a half months were devoted to relief work at the home, and 17 nurses were admitted to the Order during the year, making 37 in all.

### THE MIDWIVES BILL IN THE HOUSE OF LORDS.

The Midwives Bill passed through Committee in the House of Lords on Friday with certain drafting amendments moved by the Duke of Northumberland. Lord Portsmouth made an expiring effort in the interests of untrained women. Taking exception to the words, "No woman shall habitually and for gain attend women in childbirth unless she is certified under the Act," he contended that there were many parishes in which there was no resident midwife, and the working classes found it extremely convenient to have the assistance of women who were not trained, but who came in to assist from time to time. Were they going to prevent the working classes from getting assistance of that sort? The Duke of Northumberland answered this question by pointing out that the penalty was only in the case of women who acted as midwives habitually and for gain. "They could act occasionally for gain, as they could act habitually if they did not do so for gain." We are afraid that there may be a little difficulty as to the legal meaning of that word "occasionally," when the Bill comes into operation; but, at any rate, it is satisfactory that the sub-section which Lord Portsmouth desired to omit was retained. The amendments included a clause providing for the representation on the Midwives' Board of one person appointed for a term of three years by Queen Victoria's Jubilee Institute for Nurses.

### A LOCAL GOVERNMENT BOARD INQUIRY WANTED AT NORTHALLERTON.

The Northallerton Board of Guardians decided at their meeting last week by a majority of one—19 against 18—not to ask the Local Government Board to institute an inquiry into the following charge against the master of the workhouse:—"That he persisted in going into the workhouse infirmary ward while the nurse was dressing a male subject, which conduct was most unnecessary and undesirable." We regret their decision none the less because they assented to a motion admonishing the master, who, on the proposal of the Rev. G. T. Winch, has been told that "he had in some instances gone beyond his duty with reference to seeing to the cleanliness of the infirmary, and had also acted without discretion in improperly interfering with the nurses." The master has also been warned that in future "he must strictly confine himself to his duties in the infirmary." Admonition is all very well in some cases, but in this instance a complete investigation into the matter by a Local Government official would have been far more satisfactory. Complaints of unpleasantness between the workhouse master and the nurses at Northallerton workhouse infirmary have been rife for some time, and several nurses declare that they have left because of his interference. One of the latter states that she had to do her dressing of the patients early in the morning or late at night "to avoid the master being present," and that he claimed the right to possess the keys of all the rooms in the infirmary, including those of the nurses' bedrooms. A nurse attached to the York Home is said to have been recalled by the lady superintendent on account of his interference, and the chairman of the Board of Guardians himself went so far as to aver at the meeting on June 18th that the master's conduct had been "scandalous, outrageous, and indecent." In the face of these facts it is not fair, even to the master, to admonish him. He should have been afforded the opportunity of convincing a representative of the Local Government Board that the chairman has misjudged him; and if the chairman has not misjudged him, his continuance in his post should not have been permitted.

### THE CHANGES AT ST. GEORGE'S HOSPITAL, BOMBAY.

It is an appropriate moment to acknowledge the debt of gratitude which the community in Bombay owe to the Sisters of All Saints who, after managing the nursing arrangements at St. George's Hospital in that city for 18 years, have now resigned, feeling that their task has far outgrown its original scope, and that they are no longer able to devote themselves to it. But their gratuitous, self-sacrificing, and most valuable services through a period when it would have been difficult to have employed an adequate paid staff, cannot be too warmly praised, and we rejoice to hear that the Governor of Bombay in Council has honoured the Sisters with an expression of his cordial thanks. Bombay is not likely to forget how bravely they worked in the face of a trying climate, and of many serious difficulties and discouragements. Their places will necessarily be taken by a lady superintendent and full staff of sisters, nurses, and probationers. This, of course, means a largely increased expenditure, which will only be partly met by an augmented Government grant. At the end of May there were 36 nurses employed in the wards of the hospital and 24 on the private nursing staff. The demand for nurses for private cases is continually in excess of the number available and, happily, the fees are sufficient not only to make the branch self-supporting, but, generally speaking, to yield a surplus, which is always applied to the nursing fund of the hospital.

### HOSPITAL NURSES ON THE MERSEY.

Although the marine fête arranged at Liverpool for the week which was to have been known as Coronation week was abandoned, the invitations sent to the hospital nurses and others to view the scene on the river Mersey were not withdrawn. As it was impossible for all to be off duty simultaneously three excursions were planned, one leaving the Prince's Landing Stage about 11.30, and the others at 2 and 3.30. The Lord Mayor and the Lady Mayoress joined the first excursion. In addition to the three trips on the *Calais-Douvres*, another vessel, the *Tiger*, had on board 150 nurses from the Corporation hospitals.

### THE NURSES' HOME AT LAMBETH.

Good progress is being made in the erection of the home for the accommodation of the nurses attached to Lambeth Workhouse Infirmary. The Home, which is to contain a hundred bedrooms on three floors, thus providing a separate apartment for each nurse, is to be connected by a building to the Infirmary. Glazed brick and tile are being largely used by the builders, and both the Home and the new offices are treated in the Georgian style, with red brick and stone facings. As soon as the new buildings are completed the block now used for nurses will be devoted to patients.

### VINDICATION OF A HEAD NURSE AT NENAGH.

The inquiry before Dr. Joseph Smyth, an inspector under the Irish Local Government Board, respecting the charges made against officials of the Nenagh Workhouse by Dr. Joshua Minnitt, medical officer of the workhouse, has resulted in the entire exoneration of the head nurse from any neglect of the patients in the fever hospital. The inspector, who said that he had heard everything that could possibly be heard, declared "that from what had transpired in the evidence, the head nurse was proved to be careful and devoted." As this declaration was endorsed by Dr. Minnitt, who seems to have made general accusations hastily and afterwards to have repented of them, it can only be regretted that Sister Mary Magdalene should have been subjected to reflections for which there was no foundation. Whatever difference of opinion there may be as to the policy of employing trained lay-nurses instead of members of religious orders in hospitals and infirmaries, we strongly condemn any kind of attempt on the part of anyone to depreciate the valuable services rendered by the latter, especially in the sister island.

### "CERTIFICATED DOMESTIC NURSES."

At the annual meeting of the South Western Branch of the British Medical Association, the President, Mr. J. Delpratt Harris, in his address dealt at length with infant life. He attributed much of the defective vision so frequently found amongst children to the practice of laying children on their backs with their faces to the sky. After alluding to the lack of instinct shown in the feeding of infants he asked whether nothing could be done to bring domestic nursing into line with sick nursing? "He was aware that there were some certificated domestic nurses to be had, but that movement had begun at the wrong end, and none but the rich and well-to-do need apply. There was a distinct need of women of a higher type of intelligence and greater education to fill the post of domestic nurses, and that such women should receive certificates of education in what might be termed the professional portion of their duties, especially in correct singing of nursery songs and repetition of recitations and in nursery games." Mr. Harris was warmly thanked for his address, but his suggestion did not provoke much criticism. The movement to which he referred as already existing is, doubtless, the Princess Christian College at Kensal, Manchester, which was established in April of last year "for training ladies as children's nurses." At the end of twenty-three weeks' training, which with residence, board and laundry costs £40, a "probationer's certificate" is given. There is a danger that the use of such phrases as "certificate" and "probationer" may give rise to complications and misunderstandings. It is far from easy to get the public to realise the difference between a fully-qualified and a half-trained nurse, and we fear that an army of certificated domestic nurses would tend to make confusion worse confounded.

### A NURSE'S DOWNFALL.

A SAD career is that of Ida Mather, 37 years of age, a nurse, who at one time occupied a good position and held a remunerative appointment at Exmouth Cottage Hospital. Since she left that institution she has gone from bad to worse. In September last year she was bound over for theft at Manchester, and on December 12th, for pilfering a gold bracelet at Teignmouth, she received three months' imprisonment. She next underwent three months for a similar offence at Worcester. She came out of prison on May 12th, and on May 18th Mrs. Norsworthy, of Sidbury, left her umbrella in the hall of her residence; on her return to church Ida Mather was seen in the vicinity of the house, and it was subsequently proved that she had taken the umbrella and offered to sell it but was only successful in securing an advance of 2s. on it, after making a statement that she was expecting some money, which had not arrived, and that she would come back next day and fetch it. At the Devon Quarter Sessions the presiding magistrate sentenced her to six months' imprisonment with hard labour. We hope that when she comes out of prison this time it will be with the determination not to further dishonour her profession, but to make amends for the past.

### NURSING AT ZANZIBAR HOSPITAL.

THE Bishop of Zanzibar, who is now in England, states that connected with the hospital in the cathedral city—which is a large building used by natives and Europeans—is a nurses' home, with a large staff of trained nurses, many of whom belong to St. Barnabas Guild. It is interesting to learn that there is no difficulty in obtaining the services of a sufficient staff, although the climate of Zanzibar is so bad that the Bishop himself says that people from England taking up work there should be prepared to go out with their lives in their hands.

### AMENITIES AT A MEETING OF IRISH GUARDIANS.

AT the weekly meeting of the Derry Board of Guardians a report was read from Mrs. M. Morris, a member of the board, in which she stated that when she visited the workhouse on Saturday, June 21st, in conjunction with two other guardians, the matron handed them a report which they thought better to investigate. She proceeded to make certain allegations against a nurse and a discussion ensued, in the course of which, we learn from the *Irish News*:—

Mr. W. J. Cuthbert asked did Mrs. Morris see anything wrong.

Mrs. Morris: Don't press me that tight or I may say that I saw plenty of things wrong in the house at other times.

Mr. Gregg regarded Mrs. Morris's report as an impertinent interference with the rights of a guardian. He suggested that they should enter on the minutes that Mrs. Morris had made a report regarding a nurse and that after due discussion the letter was marked read. Mrs. Morris could then take her own course.

Mrs. Morris said her letter would go to the Local Government Board.

Mr. Hall: Shut up.

Mrs. Morris: I won't shut up, not for three long years, and years after that again.

We should have thought that, instead of indulging in recriminations, the Derry Guardians would have requested the production of the matron's report, and investigated the matter for themselves.

### NORTH STAFFORDSHIRE INFIRMARY.

THE movement for providing the nurses of North Staffordshire Infirmary with a new Home is making good progress. Towards the sum of £10,000 needed more than half has now been promised. Lord Dartmouth is to lay the foundation stone of the building in October next, and it will be creditable to the people of North Staffordshire, who seem to thoroughly appreciate the value of the infirmary, if by that date the whole of the ten thousand pounds is in the hands of the treasurer. At present the nurses sleep in the infirmary under conditions which are admittedly unsatisfactory, and the accommodation for them, generally speaking, is inadequate. The Home has therefore become a necessity.

### DEVOTION TO DUTY.

THE nurse at Knighton Workhouse Infirmary has had to resign her post in consequence of severe rheumatism following an attack of influenza. A course of treatment at Buxton and Llandrindod Hospital has not effected any substantial improvement; and the medical officer has had to report that Mrs. Evans has ceased to be physically competent for her post. At the same time he signified his opinion that "the disease from which she was suffering was due to her devotion to her duty." He added that she would never again be able to earn a living. In these circumstances, we trust that the superannuation allowance, for which the disabled nurse asks, will be on the most liberal scale.

### ANCOATS HOSPITAL.

ON Saturday last an "At Home" was held at Ancoats Hospital to recognise the efforts of the Workpeople's Fund Committee on behalf of the institution. There was a large gathering, including the Lord Mayor of Manchester, who was escorted round the wards by the matron, in company with some of the members of the Committee.

### Lectures to Nurses on Anatomy.

By W. Johnson Smith, F.R.C.S., Principal Medical Officer, Seamen's Hospital, Greenwich.

### LECTURE XXI.—THE ORGANS OF RESPIRATION.

(*Continued from page* 175).

The bifurcation or division of the lower end of the trachea into the two bronchi is not a symmetrical one. The right bronchus is larger than the left, and the upper opening takes up more of the width of the trachea. This larger opening and the deviation of the partition between the two openings to the left side serve to account for the clinical fact that a foreign body, when accidentally taken into the air-passages, passes more frequently into the right than into the left lung. The left bronchus is a little smaller than the right, and is set more obliquely, and seems in some subjects to be a direct continuation of the trachea, the right bronchus being apparently but a large branch.

Each bronchus as it passes into its corresponding lung and takes part in the lung structure divides into a large number of branches, which again sub-divide, the smaller branches, gradually losing their cartilaginous rings and plates, become at last simple tubules of thin membrane, which open into clusters of small cells called *air-cells*, varying from $\frac{1}{200}$ to $\frac{1}{50}$ of an inch in diameter. Each of these air-cells, the number of which in the two lungs has been calculated at 600,000,000, is surrounded by a very close network of capillary blood-vessels. Each terminal twig of the abundant bronchial ramification terminates in its own set of air-cells (fig. 50). Thus are formed the pulmonary

Fig. 50.—A terminal bronchial tube with air-cells.

lobules which are separated from one another by very thin layers of elastic connective or cellular tissue, and together with blood-vessels make up the whole structure or parenchyma of the lungs.

The *lungs* are two in number, one occupying the right the other the left side of the chest, the heart being interposed but pressing more on the left than on the right organ. These three organs, together with air-tubes, blood-vessels, and nerves, and some padding of fat and loose areolar tissue, fill up the whole of the thoracic cavity so that no vacant space is left. Each lung forms a mould of the cavity in which it is contained. Like the osseous framework, it is broad below where it rests on the diaphragm and narrow above where it projects a little way into the neck. The lower and broad part is the *base*, the upper part the *apex* of the lung, points to be remembered as we so often hear in ward work of tuberculous deposit at the apex and inflammatory consolidation of the base. The outer surface is large and convex, and in health is always in contact with the inner surface of the chest wall. The inner surface is shallow and excavated, and whilst the rest of the lung is quite free, this portion is attached to the heart and trachea by a so-called root which contains (1) the bronchi; (2) the branches of the pulmonary artery which carry venous blood to the lung; (3) the pulmonary veins which transmit arterial blood to the left side of the heart; (4) bronchial arteries which supply the lung tissue, and their accompanying bronchial veins; and (5) a few small nerves and lymph vessels. The anterior margin of each lung is thin and overlaps the fibrous sac that encloses the heart.

The lungs are not quite symmetrical. The left is smaller than the right, and whilst the surface of the latter is traversed by two distinct fissures running forwards and downwards and penetrating deeply into the organ so as to divide it into three semi-detached lobes, we find in the left lung but one fissure and two lobes.

The substance of the lung is soft and compressible, and when pinched crackles or *crepitates* between the thumb and finger. It is also extremely elastic, so that the lung if forcibly distended by air can be stretched out to an object about three or four times its natural size, and when relieved of this distension suddenly collapses to its usual volume driving out the excess of air. The pulmonary substance as it is permeated by air is very light, and rises to the surface when put in water. The surface of the lung is usually of a pink or light red colour in young subjects, but with advancing age, especially in coloured people, is covered by black spots and patches.

There is an important structure connected with the lungs which, though of considerable extent, is likely to be overlooked or ignored in our first inspection of these organs This is the smooth and transparent membrane which completely encloses each lung together with its root and is continued over the inner surface of the wall of the chest, on the corresponding side, the lung being thus suspended in a closed bag. This membrane is called the pleura, that over the lung being the *visceral or pulmonary pleura*, and that over the inner surface of the chest wall the *parietal pleura*. The interspace between the two layers of pleura, which, however, does not exist unless the lung is separated from the ribs by air or fluid, is termed the *pleural sac.*

Before we finish our anatomical study of the thorax we should endeavour to obtain some impression of the relative position of its contents, and of the manner in which they are packed.

The right side of the chest is almost wholly occupied by the right lung, the left side is occupied by the left lung and a portion of the heart. Each of these organs is enclosed in shut bag or sac, each of the lungs in its own pleural sac and the heart in the pericardium. The two lungs are separated by the heart and its large vessels. The two thin anterior margins of the lungs, it will be seen, do not touch one another in front of the heart, and between their thick posterior margins there is a larger gap into which the bodies of the dorsal vertebræ protrude. The small gap in front is termed the *anterior mediastinum*, the larger one behind the *posterior mediastinum.* The former is filled by loose areolar tissue, the latter, in addition to a like packing of areolar tissue, contains several large blood-vessels and nerves, and the gullet.

In the unceasing, unheeded movements which constitute what is called *ordinary* breathing about a tenth part of the air contained in the chest is expelled at each expiration, and is replaced by fresh air during inspiration, the front of the chest rising and falling from 15 to 24 times every minute. The admission or inspiration of fresh air is effected by expansion of the thoracic cavity, consequent on elevation of the ribs by active muscular movements, and by depression of the diaphragm; expulsion or expiration of air by collapse of the chest wall, by elevation of the diaphragm, and by the elasticity of the structure of the lung. The active muscular movements in healthy breathing are of the kind known as involuntary, although for a few seconds they can be arrested by the will.

In hard and deep breathing—what is called *extraordinary respiration*—we exercise our will and voluntarily bring into play several muscles of the back, neck, and upper extremities, which, under ordinary conditions, are used for other purposes. In a case of very difficult breathing, due to vitiation of inspired air, obstruction of the air-passages, or partial loss of lung function, we see the so-called muscles of extraordinary respiration in vigorous and, indeed, violent action, and the patient who is assisting their action by a sitting posture, exhausted and distressed by his unusual exertion.

In certain cases of injury and disease the mechanism of respiration is more or less disturbed. In fracture of the upper part of the spine causing paralysis of the muscles which move the ribs the breathing is carried on solely by the diaphragm, which receives its nerve supply from above the seat of injury. On the other hand, in severe injury to the abdomen and in inflammation of its lining membrane the diaphragm remains more or less at rest and the breathing is kept up for the most part by the muscles of the chest wall. The former is called *diaphragmatic*, the latter *costal breathing*.

In ordinary breathing there are also variations of mechanism in the two sexes and at different periods of life. In males and young subjects the respiratory movements are most marked in the diaphragm and the lower part of the chest; in the female in the upper part of the chest.

### Fever Hospital and House of Recovery, Cork Street, Dublin.

The following examination papers were set to the senior nurses of the Fever Hospital, Cork Street, Dublin, by Dr. J. M. Day at the midsummer examination:—

1. What symptoms point to perforation having occurred in enteric fever?
2. A child is admitted with a rash. Write down the questions you would ask the mother in order to get a correct history.
3. What complications may arise in a case of measles and how would you recognise them, and under what circumstances would you send for the physician?
4. How would you dispose of the excreta and dressings from an infectious case?
5. Describe the points to be attended to in nursing a case of tracheotomy, paying special attention to cleaning and removing the tube, ventilation, steaming, feeding, and dangers in each case.

The questions to the junior nurses are also subjoined:—

1. What do you mean by antiseptic? How can you disinfect instruments, dressings, sheets, and blankets?
2. What is blistering fluid? How do you use it?
3. Give the points of a good leech; how do you apply one and how do you stop the bleeding for its bite?
4. At what age may you give a child farinaceous food? What is the best kind to start on?
5. Describe a good vaccine mark.
6. Give the properties of good milk and the method of making sweet whey.
7. When does the rash appear in the following diseases: Measles, Small-pox, Enteric Fever?
8. How do you disinfect an empty room?

### Appointments.

Blackwall Branch Asylum.—Miss Lavinia Gillingham has been appointed superintendent nurse. She was trained at St. George's Infirmary, Fulham Road, for three years, and has since been charge nurse at the South Western Fever Hospital.

East Riding Asylum, Beverley.—Miss Frances D. Macalister has been appointed matron. She was trained in the City of Dublin Hospital. She was afterwards attached to the Royal Scottish Nursing Institution, Edinburgh, for a few years, and has been for a year one of the assistant matrons at the Stirling District Asylum, Larbert, N.B.

Epsom Workhouse Infirmary.—Miss Louisa White and Miss L. A. Cross have been appointed charge nurses. Miss White was trained at Epsom Workhouse Infirmary, and has since been attached to Swansea Nursing Institute. Miss Cross was trained at Hants County Hospital, Winchester, and has since been nurse at Stafford Workhouse Infirmary.

Fulham Infirmary.—Miss Margaret Jane Hughes has been appointed home sister. She was trained at Mill Road Infirmary, Liverpool; and the City Corporation Infirmary, Manchester. She has since been ward and theatre sister at Mill Road Infirmary, Liverpool, and ward sister at Islington Parish Infirmary.

General Hospital, Tunbridge Wells.—Miss Amy Erwood has been appointed sister of the theatre and children's ward. She was trained at King's College Hospital, London. She then did private nursing for three years on the staff of the Nurses' Co-operation, New Cavendish Street, and has since been sister of the children's ward for two years and sister of the male medical ward for one year at the County Hospital, Lincoln.

General Hospital, Weston-super-Mare.—Miss Elizabeth Rees and Miss Lucy A. Morgan have been appointed staff nurses. Miss Rees was trained at the General Hospital, Weston-super-Mare, and Miss Morgan at Chelmsford Infirmary.

Homœopathic Hospital, Birmingham.—Miss Hannah Parsons has been appointed sister of the women's and children's wards. She was trained for three years at the Royal Berkshire Hospital, Reading, and held the post of theatre sister at that hospital for seven months.

Home for Incurables, Carlisle.—Miss Ada Dixon has been appointed matron. She was trained for three years at the Guest Hospital, Dudley. She has since been night superintendent at the Guest Hospital, Dudley, with charge of the women and children's side, for 2½ years.

Monkwearmouth and Southwick, Hospital.—Miss Alice Iseline Horrocks and Miss Dorothy Van Coppenole have been appointed ward sisters. Miss Horrocks was trained at North Staffordshire Infirmary, and has since been nurse at Monsall Hospital, Manchester, and Fulham Infirmary. Miss Coppenole was trained at Cardiff Infirmary, and has since been nurse at the Hospital for Diseases of the Skin, Leicester Square, London. She has also done private nursing.

New Isolation Hospital, Wimbledon.—Miss Elizabeth Jessie Francis has been appointed sister. She was trained at the General Hospital, Ipswich, and has since been charge nurse at the North-Western Hospital, Hampstead, night sister at the City Hospital, Bristol, sister in charge at the Convalescent Home, Bristol, and charge nurse at Long Reach Hospital, Dartford.

Oxford Isolation Hospital.—Miss Margaret Gray has been appointed matron. She was trained at the Royal Infirmary, Perth, and has since been sister in charge and night superintendent at Oxford Isolation Hospital.

St. Leonard's Infirmary, Shoreditch.—Miss Florence Anne Webber has been appointed ward sister. She was trained at Hackney Infirmary for three years, and has since been assistant nurse at Fulham Infirmary and charge nurse at Brook Hospital, Shooters Hill, London.

Stockton and Thornaby Hospital.—Miss Ida Cruickshank has been appointed charge nurse. She was trained at the Royal Infirmary, Newcastle-on-Tyne, and has since been head nurse at Tynemouth Jubilee Infirmary, North Shields.

Women's Hospital, Derby.—Miss Constance Lister has been appointed staff nurse. She was trained at the General Infirmary, Leeds.

### Opening of the Henriette Raphael Nurses' Home at Guy's Hospital.

A brilliant company, favoured with brilliant sunshine, assembled at Guy's Hospital on Monday afternoon, in order to take part in the opening of the Henriette Raphael Nurses' Home, and to welcome the Prince and Princess of Wales. The Prince, it was announced, had undertaken in his capacity of President to perform the opening ceremony, and at a comparatively early hour the tent which had been erected in the Park in front of the Home was filled with visitors, the bright hues of the many coloured gowns of the medical and surgical staff, together with the light dresses of the ladies, helping to make the scene particularly striking. The period of waiting was enlivened by the strains of the band of His Majesty's First Life Guards; while the presence of a fountain, the water in its lower basin covered with water-lilies, did much to cool the atmosphere and to gratify the ears of the guests by its refreshing and continuous splash. The floral decorations were extremely beautiful, the large baskets of pale pink roses mingled with gypso-phylla and festooned with smilax which surrounded the tent were especially effective.

Soon after three the platform gradually filled up, and amongst those present were the Bishop of Rochester and the Hon. Mrs. Talbot, the Bishop of Southwark and Lady Barbara Yeatman-Biggs, Mrs Bonsor, The Lord Mayor of London and the Lady Mayoress, the Mayor of Southwark, the Mayor of Bermondsey, the Countess of Bective, Lord Revelstoke, Sir James Blyth, Sir Frederick Wills, Sir W. Cameron Gull, Lord Henry Bentinck, M.P., and Lady Henry Bentinck, Sir W. S. Church, Sir H. G. Howse, Dr. Frederick Taylor, Mr. Herbert Raphael, Sir Henry Burdett, K.C.B., Sir R. Harrison, and Sir Joseph Savory.

Arriving at the hospital punctually at half-past three, attended by Lord Crichton and the Countess of Airlie, the Royal visitors were received by the treasurer, the matron, the governors, and the members of the medical and surgical staff. By way of the colonnade and the quadrangle, the processson passed to the Home, which the Prince and Princess inspected before proceeding to the platform, where they were received with hearty applause. Her Royal Highness wore a pretty, cool figured-muslin, in shades of pink and green, with Maltese lace around the shoulders. Her toque of pink-and-white chiffon, with white ostrich feathers next to the face, worn with a white veil, was extremely becoming. The Prince, who as president took the chair, was attired in grey. The Bishop of Rochester opened the proceedings with prayers, in which he besought the Almighty to vouchsafe to all the inmates of the house peace and a patient devotion to duty.

### The Treasurer's Speech.

The Treasurer (Mr. Cosmo Bonsor) then said: It is my duty as treasurer of the hospital, on behalf of the governors, medical, surgical, and nursing staff, to welcome your Royal Highness, the President of the hospital, and the Princess, and to thank you for consenting to come to open the Henriette Raphael Nurses' Home. It is now five years since His Majesty the King—then Prince of Wales—opened the new buildings of the medical school, having previously inspired, and indeed carried through, a powerful effort to place the finances of the hospital upon an improved foundation. The action of the King in those years will never be forgotten by the present generation of governors and Guy's men, and when the time came that, as King, he could no longer retain the presidency of the hospital, His Majesty gave the best proof of his continued interest in the charity by prevailing upon the Prince to succeed him in the office. This is Your Royal Highness' first official visit, and I am expressing the feelings of everybody in conveying to you, Sir, our profound satisfaction that in times of so much stress you have been able to come to us. (Cheers.) In common with all His Majesty's subjects throughout his vast dominions, we have watched with anxiety the dangerous illness through which the King has struggled, we have admired his courage and his patient endurance of suffering, and we have realised the great strain that had been placed upon those who were nearest and dearest to him. We gratefully acknowledge the high example which the Queen and His Royal Highness have given in the time of their trouble, and we thank God that to-day we can rejoice with His Royal Highness in the fact that immediate danger is past, and that we may look forward with hope to the King's complete recovery. (Loud cheers.) It is within living memory that the nurses of this hospital lived in the basements; they were then promoted to the attics. The late Mr. H. L. Raphael took a deep interest in the hospital, and gave £20,000 for the purpose of founding a nurses' home, the only condition attached to the gift being that the home should for ever bear the name of his wife. We all regret that Mr. Raphael was not spared to see the work completed, but his family have taken and will take the greatest interest in the hospital. Guy's Hospital has been the pioneer of many improvements in the conditions under which the nurses work: the hours of duty and off-duty, so as to give the greatest relief possible; the midnight meal to the night nurses the recreation ground; and finally the Home, have all been arranged during the last five years. The King and Queen have always taken an interest in the relief of human suffering, and it is many years since Her Majesty first associated herself with the Royal National Pension Fund for Nurses, having for its object the provision of pensions for nurses, and with the kindred institution, the Junius Morgan Benevolent Fund, which provides temporary assistance for nurses who are disabled by sickness or accident. We all rejoice in the kindly interest which the Royal Family have shown towards a hard-working profession, and I am especially rejoiced to state what an enormous gratification it is to the nursing staff that the Princess of Wales has come with His Royal Highness to identify herself with the admirable scheme which has been brought to successful completion. (Loud cheers.)

### The Prince of Wales's Speech.

The Prince of Wales, who was received with loud cheers, said:—Before I allude to the object for which we are met together to-day, I am sure that all who are here will join with me in expressing our feelings of unbounded thankfulness to God for the merciful recovery of my dear father, the King. (Cheers.) And I wish to take this first opportunity which offers itself to say how His Majesty the King, the Queen, and the whole of our family have been cheered and supported during a time of severe trial by the deep sympathy which has been displayed towards them from every part of the Empire. (Cheers.) Speaking before the authorities of one of our leading hospitals, I should like to say that we who have watched at the sick bed of the King fully realise how much, humanly speaking, is due to the eminent surgical and medical skill, as well as to the patient and highly trained nursing which it has been His Majesty's good fortune to enjoy. (Hear, hear.) So it seems most fitting that one of the first public ceremonies that the Princess and I should take part in since the King's serious illness, should be to open this beautiful Home for nurses within the precincts of this great hospital, which we have just had the pleasure of inspecting. It is only in comparatively recent times that the *rôle* of the nurse in the sickroom has been fully recognised. But are there not many here who, like myself, will throughout our lives, remember with the deepest gratitude the soothing comfort, indeed, I may say the blessing, of efficient nursing? Once the value of its work was organised, nursing has been more and more looked upon as a proud and honourable career. The recent war has shown us what a benefit the country derives from having in its civil hospitals a reserve of nurses available for service in the field. (Hear, hear.) We know what splendid work has been done in South Africa by the nurses largely drawn from the training schools, such as exists in this hospital, and I am sure the thanks of the nation are indeed due to the general hospitals who sent their best nurses to cope with the serious difficulties with which the military medical authorities were at one time confronted. Recognising, therefore, the high and indispensable position which nurses occupy, the least that can be done for them is to provide the comforts of a home where they can enjoy rest and relaxation after their hours of arduous and self-sacrificing work. It was one man who, holding this view, gave practical reality to it by the munificent gift already referred to by the treasurer, for the purpose of establishing this home which we to-day inaugurate; and I am glad, as your President, to congratulate Mr. Raphael, who I am happy to think is one of our governors, upon the consummation of the work of which his father was the founder, and which for all time will bear the name of his mother. (Cheers.) Ladies and gentlemen, I wish to thank you for the kind reception which you have given to the Princess and myself, and I can assure you that we both feel, in coming here to-day, that we have only fulfilled a very pleasant duty. I thank you also, Mr. Treasurer, for all your kind allusions to the King and Queen. It is not too much to say that the King, on his accession, laid down with great regret his work as President of this hospital, and also of the Hospital Fund for London, which he founded, and which still bears his name. I am sure you all know His Majesty continues to take the keenest interest in the progress of this fund, and in the excellent work which it has done, and he looks forward to the still greater work which the organisation now created is ready to carry out whenever the increased funds for doing so are available. I have now much pleasure in declaring the Henriette Raphael Nurses' Home open. (Loud cheers.)

### Other Speeches.

Lord Aldenham, in moving a vote of thanks to their Royal Highnesses, alluded to the year when he was President of the hospital, and mentioned that, from a monetary point of view, he fell upon evil times. But then the King came, and at once matters began to mend, and, as Lord Aldenham believed, will under the presidency of the Prince of Wales continue to go on improving.

Mr. Herbert Raphael, who as soon as his identity was fully realised by the audience, was loudly applauded, seconded the vote. He referred to the fact that, although this red-letter day in the annals of hospital work was naturally a great source of pleasure and satisfaction to him and his family, it would always possess for them all a solemn note, for not only had she who had spent her life in helping the sick poor, and whose work his father had sought to commemorate, passed from their midst, but his dear father had also been called away before the completion of the Home in which he took so keen an interest.

Mr. Bonsor then declared that the vote was carried by acclamation, and shortly afterwards the Prince and Princess left the grounds, an enormous crowd of the poorer inhabitants around Southwark having assembled to cheer them as they drove off.

### Inspecting the Home.

Most of the visitors, after partaking of tea, inspected the Home, and many were the exclamations of admiration and surprise heard on all sides. The principal rooms were beautifully decorated with flowers, the tall white lilies in the general sitting-room, which is a magnificent apartment with its polished floor, its eastern rugs, its handsome furniture and its cosy armchairs, showing up in strong contrast to the rich colouring of the surroundings. The Sisters' sitting-rooms are comfortably furnished, and the dining-hall, capable of accommodating 170 diners at once, each table covered with a white tablecloth and adorned with large bunches of pink roses, was much discussed. As yet the frescoes on the wall are only sketched in, not painted. The 213 bedrooms are all similar in design, and are furnished with a bed, chair, table, combination chest of drawers and wardrobe, and a porcelain basin fitted with hot and cold water supply. But the greatest crowd was always to be found passing up and down the narrow staircase to the swimming-bath. The clear cool green water looked extremely inviting in the heat of Monday, and the whole place, with its clean white walls festooned on all sides with flowers and wreaths, was a refreshing spot in which to linger. A visitor nurse from another hospital was heard to remark to a Guy's probationer, "This is the only thing I envy you." And the handsome supplemental present of Mr. H. H. Raphael and his brother, Mr. Walter Raphael, is certainly one of the most notable features of the new building. A detailed and illustrated account of the Home appeared in The Hospital of April 19th.

### Fete in Aid of St. George's Hospital.

A charming spectacle was witnessed at the Royal Botanic Gardens on Friday last week, the occasion being a fête in aid of St. George's Hospital. It had been feared that owing to the King's illness this event would have to be postponed, but reference having been made to the Prince of Wales, his Royal Highness directed the fête to be held, as it was in aid of a charity. The weather was everything that could be desired, and the beautiful Gardens looked their best, a wonderfully festive appearance being imparted to them by a multitude of gorgeous triangular flags from Northern China. The proceedings commenced at 1.30, and by 3 o'clock the Gardens were filled by a large and fashionable company. Both luncheon and tea were served on the grounds in large marquees. The entertainments provided were of a varied and attractive description, the bands of the 1st Life Guards and the Royal Artillery rendering selections at intervals during the afternoon and evening. Variety shows were held in one of the palm houses, when a number of well-known artists kindly gave their services, Mr. Dan Leno being, however, unfortunately prevented from attending. There was also a pastoral play, consisting of the third act from "Othello," by permission of Mr. George Alexander. The entertainment which proved the greatest attraction was the "haka" or war dance performed by the Maoris of the New Zealand contingent. The war dance took place in a hollow, upon the slopes of which stood rows of interested spectators. The Maoris, who were all particularly fine-looking men, under the direction of a leader picturesquely attired in native costume and plentifully bedaubed with war paint, went through a weird and striking performance accompanied by curious explosions of hoarse shouts. So popular was this war dance that it had to be repeated later in the afternoon, but at the second time the men were attired in their khaki uniforms, and the repetition was necessarily less picturesque. The Royal Family were represented by Princess Henry of Battenberg, accompanied by her children, Princess Christian, Princess Louise of Schleswig-Holstein, and Princess Frederica of Hanover. King Lewanika, two or three of the Indian Princes, and the Agents-General for the Colonies were also present. Altogether between 1,500 and 2,000 tickets were sold, so that when the proceeds from the entertainments and other sources are taken into account the hospital should benefit materially by the fête.

### An Interesting Experience in the Philippines.

BY AN ENGLISH NURSE.

I am afraid I must plead guilty to having always possessed a love of novelty and excitement, and although I thoroughly enjoyed the period of my training—commenced at the Portsmouth Royal Hospital, and completed at St. Bartholomew's Hospital—the completion meant to me freedom to choose my future life. A soldier's daughter, what better could I wish than to become an army nurse? First, the home military hospitals, then the foreign, and then, who knows, perhaps the field! I sent in my name and credentials, and was found eligible, but the long list of names in front of mine, awaiting vacancies, discouraged my restless spirit, and I gave up the idea. I was offered the post of night superintendent at one of the large North American hospitals, which I gladly accepted, and it was a step I have never regretted, for, strange to say, it was to be the means of my obtaining my heart's desire. At the end of my third year's service, war broke out between America and the Philippines, and I could not resist another effort to become an army nurse. This time I was successful, and after spending some months on the staff of one of the military hospitals in America, I was drafted off to Manila, where I have now been nursing for two years at one or other of the military hospitals in or near Manila, and have been most happy in my work amongst the soldiers.

### The First Civil Hospital.

I received promotion, and at the time of writing, am chief nurse in the first civil hospital in the Philippines. It is a 100-bed establishment for the employés of the civil government, and there are many ex-soldiers, especially of the volunteers, who have stayed out here and taken civil positions. There are only ten female nurses, and ten male nurses, who have a home of their own adjoining the hospital. We have our home some little distance from the hospital but as conveyances are provided for us, we find it very nice to be away from the seat of our work. Like all houses in the Philippines, ours is very airy and large. We have, too, a lovely roof-garden, and a verandah all round the house. Certainly it is an ideal place for a nurse to live in, and the work in the hospital is not heavy, for all the hard work is done by the Filipinos and male nurses. But I must not be tempted to fill too mnch space with an account of the hospital life, as I am anxious to tell my English friends of a most wonderful and perfectly fascinating trip which fell to my lot in April of this year.

### A Ten Days' Trip.

In connection with our civil hospital, the committee have opened up a sanatorium for the convalescents in the province of Benquet, and I was asked by the doctors to take a ten days' trip with the superintendent, to see how things were progressing, and if I liked the place to remain there for three months, or permanently if I wished. So with my usual alacrity for novelty of any description, I packed a small valise and hand-bag with actual necessities. Our way lay by train from Manila to Dagupan, then in an ambulance waggon through the country, and after that by native bull carts—the most terrible form of transportation, as they practically take an hour a mile. We were four and a-half days on our journey and never in my life did I enjoy anything so much. It was the most interesting, romantic and marvellous journey that I have ever taken. I am not a very excellent horse-woman, and to have to ride along a mountain trail, hardly wide enough to let a man and horse go through, and at a height of 5,000 feet above sea-level, was a difficult but at the same time exhilarating experiment.

### A Native Breakfast.

After one day and a half trip up to Naguillan we stayed the night with a Filipino chief, in a Nippa hut, and had a native breakfast, which I could scarcely swallow, but it was the best to be had in the land. The superintendent and I started on our mountain ponies, and after a short ride we arrived at the foot of our tremendous climb—it was simply up and down hill through the most gorgeous scenery, and our guide was a little naked Igonotè boy, who of course knew not a word of English. At 12 noon we camped beside a natural spring in a most charming spot, and had a lunch of dried beef and cherries, simple, but satisfying. About 2 p.m. we met the Governor of Benquet, who had come purposely to meet us, and wanted to take us along a new trail, so that we could meet some Igonotè chiefs. Our cavalcade by this time amounted to seven—including the Governor's secretary, an Igonotè chief and guides. The ride was truly terrible. In many places my pony had to be led, for I was too frightened to cross. At 9 p.m., in the most dreary, lonely spot, we arrived at chief Antonio's house. We found a low wood house, almost bare of furniture, and innumerable natives with torches, wearing no clothing save loin-cloths, rosary on neck, looking out for us.

### Native Wine and Weird Music.

We had to sit around on the ground, and drink the health of everyone in native wine, which I must confess was very strong, and left me with a distinctly suspicious feeling in my head; but I was the first white woman over this trail, and such an object of curiosity to the natives, that I was obliged to drink to all toasts, and they kept coming over to look at me, and to feel my dress. Then, although I was dead tired, we had all to sit up and listen to some weird music and singing by the Igonotès. It was so uncanny, that I should have been a little frightened but for the close presence of the Governor and the superintendent. Bedtime came at last, and only two bedrooms! The whole of that company had to sleep in one room, whilst I, like a queen, occupied the other. My bed was simply a bamboo bench with a mat on it, and not another piece of furniture in the room.

### Arrival at Bagnio.

Next morning we started off, and had to climb 2,000 feet higher. We had lunch at another Igonotè chief's house, and then made our last grand effort to reach Bagnio. During the ride the brambles tore my riding skirt. I was black and blue with bruises, lost my hatpins, then my hat. Also my hair was torn down by the branching brambles, and to crown all a mist came over the mountains and I had to trust to my pony entirely—which made me very nervous, but the Governor rode in front and the superintendent behind, which gave me confidence. At last we reached Bagnio, and I was so tired that I had to be lifted off my horse and carried upstairs.

### The Sanatorium.

We had ridden 47 miles that day, but the goal was worth it. Our little sanatorium is only a small wooden building, but the grounds and pine forests right around are grand beyond description, and I was so charmed with it that I at once decided to remain, at any rate, for the three months. It is lonesome but I enjoy it. My pony is provided by the hospital, and I have my own saddle, and after work, with my Igonoté, who rejoices in the name of "Kit-Kat," as my guide, I ride over the mountains until supper-time with no ordinary human beings to talk to—only the mountains and the sky—and I can always enjoy such a situation. At present our full complement consists of only two men and myself. Next week one more nurse and two men will be added, and ten patients are to be carried up on mountain chairs, so we shall be a happy little family.

### How to Manage a Severe Case of Rheumatic Fever.

EXAMINATION QUESTIONS FOR NURSES.

The question was as follows:—"How would you treat (so far as a nurse's province extends) a severe case of rheumatic fever?"

### First Prize.

In nursing a case of acute rheumatism, patient must be kept between blankets and must wear a flannel nightdress, as warmth is of utmost importance. If there is pain in the joints, such as the knee or elbow, hot fomentations sprinkled with A.B.C. liniment may be applied, or the part may be wrapped in cotton wool, previously warmed and kept in position by a bandage. The lower limbs may be protected from pressure by a cage. As the skin must be encouraged to act freely, patient should be washed all over night and morning with warm water and soap. This is also beneficial in removing the sour disagreeable odour of perspiration. Every precaution must be taken to avoid chill. A recumbent position must be preserved throughout the illness. Any complaint of a feeling of oppression over the heart or palpitation must be reported to the doctor. Bedsores may be guarded against by frequent rubbing with camphorated spirit. Diet should be restricted to milk, barley water, and alkaline drinks during the acute stage, gradually passing on to milk puddings, fish, and white meat. Large quantities of fluids should be given to assist in throwing off waste matter. If patient is having salicylate of soda, the nurse must report any complaint of deafness, giddiness, or ringing in the ears. Great care must be taken in avoiding exposure to cold, even after convalescence.—"Nurse Ina."

### Second Prize.

A patient suffering from rheumatic fever should wear a loose flannel gown and sleep between blankets, rather thin old ones are best. The gown and the top blanket at any rate should be changed night and morning, dried in the open air (when possible), and well aired before being put on again. Clean ones should be used as frequently as circumstances will permit. A draw sheet and mackintosh may be used if it seems best, but a large mackintosh next the mattress is better in these cases. Rheumatic fever patients sweat very freely and require sponging with hot water at least twice a day. The utmost care must be taken to avoid a chill. All clothing must be put on warm, even to the wool or flannel bandages, usually wrapped round painful joints or limbs. Shut the window and lock the door during the sponging. Temperature, pulse, and respiration should be recorded on a chart every four hours, and it is best to save the urine and measure it once in 24 hours. Diet consist at first of milk only, which may be given hot or cold, diluted with lime, barley, or soda-water, or flavoured with tea, coffee, or cocoa. At least ℥v should be given every two hours, if the patient can take it. Broth, beef-tea, or arrowroot will make a little change if the doctor will allow them. As convalescence advances, soup, milk-pudding, jelly, blancmange, toast, fish, and chicken may be gradually added to the dietary, with the doctor's permission. The suffering in moving the affected joints is quite indescribable, therefore be very very gentle during the necessary sponging, etc. Pillows of different sizes are most useful to put under the more painful joints now and then to ease the pain. Everyone coming in and out of the room must be cautioned against touching the bedstead, banging the door, and treading heavily, for either of these things shakes the patient a little, and causes pain. The parts liable to become sore must be rubbed with methylated spirit and well powdered with zinc and starch, or boracic powder. If salicylate of soda is given, watch for deafness, noises in the ears, and "wandering." Report such symptoms to the doctor, but if he be away and they increase, lessen the dose or even omit it if necessary. It is well to ascertain his wishes beforehand regarding this. It is dangerous to let a rheumatic fever case sit up, as the heart is mostly somewhat affected, and a nurse can hardly tell how much.

Aunty.

### A Good Standard Reached.

Taken as a whole the answers this month are good, though I would fain see the effect of more thought and also more care in writing. May I say how glad I am that "Magdalene" has taken my strictures on her style in good part, and that she has sent in a very good paper well expressed. A nurse who has the common sense to take a correction without offence and at once to try and profit by it, has capabilities for much.

Several papers are nearly as good and in some respects better than "Nurse Ina's" but she wins the first prize because she realises the necessity of a real, honest soap and water head to feet washing twice a day, in distinction to sponging, which is quite a different matter; only two other nurses mention this *cleansing* washing, and the omission is rather serious, and betrays a want of observation and thought. Mere sponging does very little towards cleansing and the sour smell pertaining to rheumatic fever is hardly improved by it. Moreover, the alkali found in most soaps is beneficial.

"Aunty," the winner of the second prize, does not mention this cleansing process, but she is a thorough-going person all the same, for she goes the length of locking the door when sponging her patient, lest an officious assistant should open it and let in a draught. She also speaks of the extreme need of care not to shake the room or jar the sufferer in any way—a most necessary caution, for the agony thus caused can hardly be estimated by the healthy.

### Honourable Mention.

This is gained by "Daylight," "Paddie," and "Cautious."

A very good paper was sent in by "Marguerite," but as it arrived five days after the limit of time allowed for competition, no notice could be taken of it.

### Treatment that is Risky.

Several competitors say that where there is an exceptionally high temperature they should *cold* sponge. Pray do nothing of the kind without express orders from a doctor. Having regard to what is frequently the condition of the heart in such cases as are now under consideration, you would be assuming far too serious a responsibility. Never attempt to enter the medical man's province and to take on your shoulders grave burdens that belong to him alone. To know her own limitations is one of the marks of a competent nurse.

There will be no further questions till October.

The Examiner.

### Rules.

The competition is open to all. Answers must not exceed 500 words, and be written on one side of the paper only. The pseudonym, as well as the proper name and address, must be written on the *same* paper, and *not on a separate sheet.* Papers may be sent in for fifteen days only from the day of the publication of the question. All illustrations strictly prohibited. Failure to comply with these rules will disqualify the candidate for competition. Prizes will be awarded for the two best answers. Papers to be sent to "The Editor," with "Examination" written on the left-hand corner of the envelope.

N.B.—The decision of the examiners is final, and no correspondence on the subject can be entertained.

In addition to two prizes honourable mention cards will be awarded to those who have sent in exceptionally good papers.

### To Nurses.

**We invite contributions from any of our readers, and shall be glad to pay for "Notes on News from the Nursing World," or for articles describing nursing experiences, or dealing with any nursing question from an original point of view. The minimum payment for contributions is 5s., but we welcome interesting contributions of a column, or a page, in length. It may be added that notices of appointments, entertainments, presentations, and deaths are not paid for, but that we are always glad to receive them. All rejected manuscripts are returned in due course, and all payments for manuscripts used are made as early as possible after the beginning of each quarter.**

### Everybody's Opinion.

[Correspondence on all subjects is invited, but we cannot in any way be responsible for the opinions expressed by our correspondents. No communication can be entertained if the name and address of the correspondent are not given as a guarantee of good faith, but not necessarily for publication. All correspondents should write on one side of the paper only.]

### THE FOOD QUESTION.

"AN ASSISTANT NURSE" writes: Having read with interest "E. S. E.'s" comment upon poor "M. A. B." I have come to the conclusion that "E. S. E." is not a nurse, but an official. I am not a lady of independent means, neither am I one of the servant class, but I should like to know what lady would be content to sit down to half a bloater for breakfast twice a week. "E.S.E." thinks that bread-and-butter for tea every day is very good, but a general servant nowadays turns up her nose at that. I am sorry to say that in the hospital where I have been for some time the cooking is exactly as "M. A. B." describes. It is no unusual thing for half the nurses to get up and walk out of the dining-room, one look at the dinner being quite sufficient. Of course, if "E. S. E." is content with such a bill of fare, I can only draw my own conclusion as to what her idea of a lady is. Might I suggest that "M. A. B." is one of the gentlewomen and not the servant class. I think if some of our nurses would carry their food to the committee they would be rewarded for their trouble.

### NIGHT NURSES ON HOSPITAL SHIPS.

"BE JUST" writes: It is much to be regretted that a list of the fancied hardships borne by the nurses on the M. A. B ships should have found its way into THE HOSPITAL, instead of being laid before the medical superintendent or matron who are always willing to meet us as far as lays in their power. From the tone of the complaints, I should imagine our informant was at some time or other deputed to carry over the offending joint, but there is great reason to doubt if it ever weighed 20 lbs., and if so, I do not think that would be an unreasonable weight for any healthy woman to carry 300 yards. Also there is nothing very unpleasant in the journey to and fro on the tug *Angelina*, as she is fondly called. I feel sure many will agree with me in thinking it far better for night nurses to take their meals and sleep on shore, where everything is fresh and quiet, to being on board where there is bound to be more noise. With regard to a nurse being refused a cup of tea if she feels ill or unfit to come to the dining-room, it is quite untrue; if the night superintendent is sure she would be better for the extra rest, she will always arrange for another nurse to take her what is required; it is when these privileges are abused, as they often are, that the need for stopping them arises. Also, why should there be any objection to taking the patients' suppers in. As we change at 7 P.M. it would be impossible for the day nurses to give them, unless they began at 6 P.M., which is far too early for patients to sup considering breakfast is not till 7 A.M. next morning. Far better to have something to do than for the want of it go into the ward kitchen and gossip and criticise the ones whose authority they are under, be they ward matrons or charge nurses. I consider the nurses have small cause to complain, especially the assistants. I have worked in many general hospitals, large and small, but have never met with as much consideration as is to be found under the M. A. B. We have good salaries, good food, liberal off-duty time, and many other comforts too numerous to mention, and if the day ever comes when the medical superintendent or matron refuse to help us, well, we can always go to the committee.

"CHARGE NURSE" writes: Respecting the remarks in THE HOSPITAL on "Night Nurses on the Hospital Ships" I should like to be permitted to state that for nearly three months last year I held the post of charge night nurse in one of the hospital ships, and, despite the fearful strain caused by the rush of cases as the epidemic increased daily, have seldom, during many years' experience of different hospitals, been in an institution where the comfort and convenience of the nurses was studied to such an extent as on the ships. Whether more stringent rules have had to be enforced during the last six months, owing to the abuse of privileges allowed by unthinking assistant nurses, I cannot say; but in my time we could certainly "have a cup of tea" in our bedrooms, and, personally, my greatest pleasure was, on coming off duty at 8 A.M., to spend the time before retiring to bed at noon; sitting on deck in the sunshine (where seats with awnings were provided for the nurses' use) watching the ships, or taking a brisk "constitutional" up and down with a friend. Indeed, matron used to encourage us to get all the air we could, either on deck or across in the fields on shore, where anyone who was interested in botany or kindred studies could find treasures galore. As to the over-worked individual who "has to carry meat on a dish . . . for the appalling distance of 300 yards" in "all sorts of weather," and then, after such a fatiguing trial of strength, is actually forced "to carry round the patients' suppers," I am afraid the poor thing is not quite strong enough for the duties expected by the M. A. B. from their assistant nurses and I wonder how she would survive the "pro-ing" that I and my fellow charge nurses on the ships uncomplainingly performed during our training. She—I presuppose that the complaining correspondent is an assistant nurse—does not mention that every nurse on the ships is provided with a mackintosh cloak, thick shawl, and sailor hat as well as shoes, in order that she shall be well protected from the weather when crossing the decks or passing from ships to shore. She also omits to say that the "suppers," the carrying round of which appears to be such a grievance, can all be given out in five minutes by means of methodical distribution and the use of a tray!

### AN APPEAL FROM A PRIVATE NURSE.

"M." writes: Could not "Nurse Marguerite" arrange her hours differently? I always manage, when there are two nurses, to go on from 2 till 2. The nurse who goes on at 2 A.M. goes to bed at 6 P.M., after having her bath and taking a cup of cocoa, with bread and butter or biscuits, as she is going to bed, at 2 A.M. If a female patient, she goes on duty in her dressing-gown, bringing her clothes in with her; the other nurse having a cup of tea and some cold meat, eggs, tongue, or other light food ready. If a male patient, she would require to be called a few minutes earlier to dress. The other nurse retires at once, generally having her breakfast in bed between 9 and 10, has her bath, gets up and goes out till 1.30, when she has her dinner, being ready for duty at 2 P.M. Not being, I suppose, a thoroughly up-to-date nurse, I have my dinner sent up before going off duty, eating it according to the case in the patient's room, or in the doorway. This saves worry to the family, and one can get away at once after being relieved.

"A LATE HOSPITAL SISTER" writes: "Nurse Marguerite" should alter the hours on duty. I have been private nursing for many years, and when I have another nurse to share the work with me I arrange to be on from 2 P.M. until 2 A.M. By this means we both get a little real night in bed, and I find it more comfortable all round. At 2 A.M. I have tea ready for the night nurse; this she has with whatever has been saved for her from the late dinner or supper. I then go to bed, and night nurse calls me at 11 A.M. When I have had coffee, tea, or cocoa, bread and butter, etc., I then go out for a walk. At 1 or 1.30 P.M. I have had dinner, nurse going down first, and then I relieving her. At 2 P.M. she goes off duty, has a walk, comes in for tea, and can be in bed by 5 P.M., and by so doing can have eight hours' good rest. I, of course, get the same.

"A. E. L." writes: May I, through THE HOSPITAL, recommend a little common sense in "Nurse Marguerite's" case, in preference to "philanthropic agitation," which, I think, would probably result in making the nursing profession a laughing-stock? Surely it is possible for "Nurse Marguerite" to take her two hours' off-duty, as many nurses have to do, before 9 A.M. She cannot require the whole twelve hours for sleep, and a good walk or bicycle ride when the day is young and fresh would do much towards giving her a more cheerful view of life than she apparently takes at present. Or could she not change with the night nurse for a time and so have her days to herself? Or she might even change the time of going on and off duty. I have had experience of chronic cases—sole charge of one for three years—and am of opinion that these arrangements can easily be made by the nurse herself, and carried out without friction or encroaching upon working hours, if sensibly and pleasantly managed.

---

PRIVATE NURSING HOMES.

"E. F. W." writes: In your last issue there is a letter about the above from "H.," whose experience has evidently been unfortunate, and whose word I do not doubt. But in justice to others I feel that I must write and tell of the other side. Through advertising, I heard of a home in the Midlands, and after more than two years' stay there I think I am qualified to judge. It is the private property of and kept by two sisters—educated ladies and fully certificated nurses—is well furnished, beautifully clean, ordered as a lady's household should be, with food good and varied, and moderate fees. There are nine or ten patients, the nurses working under the sisters' supervision; and as they are constantly amongst their patients, nothing is done without their knowledge. I, as a helpless cripple, cannot speak too highly of their kindness, care, and patience. As to Government inspection, they would not fear it, their objection being that it would destroy, at least in part, the feeling that it is a real home, which they wish to maintain.

"D. H." writes: I have nursed for many years in one of the largest nursing homes in London and now have one of my own and am personally acquainted with the management of several others. My experience convinces me that the sweeping assertions contained in "H.'s" letter apply only to a most exceptional establishment. One accusation brought against nursing homes is certainly true—the fees are high, though not exorbitant in comparison to the necessary outlay. This very expense acts as a safeguard; it keeps out the class who notoriously confide themselves to unconscientious quack doctors. The inmates of nursing homes are usually patients of provincial practitioners who come to town to be treated by specialists. They are almost invariably sent by the specialist himself to the particular home which he knows and patronises. Often the patient's recovery depends as much upon the nursing as upon the specialist's skill, and it is therefore unlikely that a surgeon or physician would risk the life to his patient and his own reputation by entrusting his case to the care of nurses whom he knows to be incompetent or does not know at all. Again, a certain amount of work comes to a home from the recommendation of past and grateful patients. I know enough of the excellent management of many homes and of the keen competition to affirm that such a home as "H." describes would necessarily "go to the wall" from lack of support. There is usually for such patients a heavy fee to be paid first to their doctor. A middle-class patient could not afford to spend months in a nursing home and those of the wealthier classes would not find the life sufficiently entertaining to induce them to do so. My experience is that nurses infinitely prefer short to longer cases; hence the popularity of surgical *versus* medical homes. I believe the fault, if there is one, to be rather in the tendency to hurry off patients too quickly. As to the inefficiency of nurses, some homes do follow the system of training their own probationers, but in this case one usually hears the beginners themselves complain that no responsibility is given them, and that all work but the most elementary is performed by the sister in charge of them. Most matrons are far too anxious to retain the support of their particular doctors to allow their cases to be neglected by "untrained, inconsequent girls." As to registration, the majority of London nursing homes are situated on one certain estate. The owners of this estate require nursing homes in the main streets to be licensed. This is not absolutely necessary in the case of the side streets, but homes opened without a license run the risk of compulsory closure at any time after notice from landlord or ground landlord. The license contains provisos as to good management, and is only granted to those who can supply satisfactory evidence as to respectability and competence to the ground landlord. I venture to think that this license, together with the high standard of nursing required by London doctors, is sufficient to exterminate those extraordinary homes which "H." has apparently met with.

### Annual Meeting of the Colonial Nursing Association.

THE sixth annual meeting of the Colonial Nursing Association was held at Kensington Palace on Wednesday afternoon. Owing to the illness of the President, Earl Grey, the Vice-President, the Earl of Westmeath, took the chair, and there were present, among others, Princess Henry of Battenberg, Viscount and Viscountess Knutsford, Lady Balfour of Burleigh, Mrs. Chamberlain and Mrs. Endicott, Mrs. Francis Piggott, Mrs. Ernest Debenham, Hon. Sec., and Sir West Ridgeway.

The Earl of Westmeath, in opening the meeting, read a letter from Earl Grey expressing his great regret at being unable to attend, owing to his recent illness. His Lordship then went on to say that the report (a summary of which appeared in THE HOSPITAL of last week) would be taken as read, and needed very little comment. They all knew the history of the Association, and how it owed its existence to Mrs. Piggott, who had been so much struck with the bad arrangements made for nursing in the colonies, and who had devoted so much time and labour to the improvement of this state of things. Mrs. Chamberlain also took a deep interest in the Association, and they would all remember how earnestly she had pleaded for £5,000 in order to put matters on a proper footing. They had at last got that £5,000, but they had not yet got all that they desired, as would be seen from the report, which showed how fast the Association was growing, the number of nurses employed having gone up in the past year from 29 to 41. Lord Westmeath then read some interesting letters from nurses abroad, one in particular describing the terrible state of things at Georgetown after the volcanic eruption.

Sir John Stirling Maxwell, M.P., who moved the adoption of the report, said that the work of the Association appealed to everyone who had been touched by the wave of Imperial feeling that had swept over the Empire, and that no better means could possibly be adopted for the closer knitting together of the Empire than the establishment of such an Association as this, which sent out nurses to the most distant parts of our possessions.

Mr. Hugh Clifford, who was the next speaker, drew a pathetic picture of the miseries he had undergone when lying ill in the Straits Settlements, attended only by Malays; and he demonstrated with much vigour how entirely different was his experience when, during a later attack of illness, he was waited upon by nurses sent out by the Association. There was one suggestion he had to offer, and that was with reference to the nurses' hours on duty: when he was under their care they had three shifts of eight hours each, and he strenuously maintained that in the tropics eight hours at a stretch was far too long for women to be on duty, and recommended, if it was possible, four shifts of six hours each.

Sir Charles King-Harman testified to the excellent results obtained by the Association in Sierra Leone. He had been connected with the Association from the start, having been resident in Mauritius in 1895, when Mrs. Piggott first arrived, and saw how sufferers were neglected or mismanaged owing to the absence of trained nurses, and he joined in the general satisfaction that was felt at the splendid results of her efforts.

Lieut.-Colonel Montanaro, commandant of the Aro Expedition, and Colonel Man Stuart, who commanded the lines of communication during the last Ashantee expedition, both bore emphatic testimony to the inestimable value of the services rendered by the nurses who accompanied both expeditions.

Miss Louisa Stevenson pleaded for a more prolonged training for the nurses.

Votes of thanks to Princess Henry of Battenberg and the Earl of Westmeath were passed, and the crowded meeting separated.

### Echoes from the Outside World.

### The King's Recovery.

On Saturday morning the King's doctors signed a bulletin stating that they considered the King "to be now out of danger," and signifying that the evening bulletins would therefore be discontinued. Since that day excellent progress has been maintained, and on Monday the Queen immensely gratified a number of old people dining at the Town Hall, Chatham, who sent hearty congratulations to her on the recovery of his Majesty, by sending them through the Mayor a message, "The Queen thanks you for your kind sympathy. The King is progressing most favourably."

### The King's Dinner to the Poor.

Half a million of guests were entertained in London by the King on Saturday. At all the 700 centres where the guests had assembled, the morning bulletin from Buckingham Palace, stating that the doctors considered the King now out of danger was read, together with the message from His Majesty to the Lord Mayor of London, communicated by Lord Knollys of Caversham. "I am commanded by the King to inform your lordship that His Majesty and the Queen had intended visiting some of his Coronation dinners to-day, and he deeply regrets that his illness prevents their doing so. The King has deputed members of his family to represent him at as many of these dinners as possible. I am further commanded by the King to express his hope that his guests are enjoying themselves, and are passing a happy day." The Prince and Princess of Wales were indefatigable in visiting the feasters, arriving at Fulham about 12.30, where they got down from their carriage and walked up and down the tents, then returning to York House for lunch and starting off in the afternoon to Poplar. Hence they went to Victoria Park, then to Hackney, and last of all to the People's Palace. They were received everywhere with vociferous cheers. The Duke and Duchess of Connaught went to Holborn, Finsbury, Shoreditch, and the Guildhall; the Duke and Duchess of Fife to Westminster, Chelsea, and Battersea; Prince and Princess Charles of Denmark to Southwark, Lambeth, and Camberwell; Princess Louise and the Duke of Argyll to Olympia, Kensington, and Paddington; Princess Christian to Marylebone, St. Pancras, and Islington; and the Duchess of Albany to Bermondsey, Deptford, Greenwich, and Lewisham. The dinners, of course, varied with the ideas of the different committees, but in most places it consisted of cold beef, mutton, and ham, hot potatoes, plum pudding, with tobacco and cigarettes for the men, and sometimes chocolate in addition. In some places beer or cider was given, in others only non-intoxicating liquors were supplied. At Paddington an old couple were introduced to the Duchess of Argyll who had both been present at Queen Victoria's coronation dinner. They were boy and girl then, and had been married 50 years. The old man carried his long-cherished invitation card of 1838 in a frame under his arm.

### The Queen's Tea to Maidservants.

The Queen's teas to maids-of-all-work commenced on Monday, and there were large gatherings at Holloway and Lewisham. Each guest received, after a substantial meal, a red, white, and blue buttonhole and a box of chocolate. The latter bore the simple inscription, "Coronation Souvenir from Queen Alexandra, 1902." Specimens were also shown to the guests of the brooch which each of them is to receive as soon as the large order can be executed. On the face of the pretty brooch is inscribed the Royal monogram, "A. R." and "1902," and on the back the words "From the Queen." The 600 tea-drinkers at Holloway sent a telegram to the Queen thanking her for her gracious thought of them. On Tuesday 750 "general" servants were entertained in the great hall of Christ's Hospital, and, at the suggestion of the Bishop of London, they sent a telegram of thanks to the Queen.

### Presentation to Prince Edward of Wales.

On Friday last week an interesting presentation was made to Prince Edward of Wales. The children of Paddington had a fête on Thursday, and as the Prince and Princess of Wales were unable to be present they consented to receive a deputation—consisting of one child from each of the 29 schools—at Marlborough House. Amongst those who attended were Lord Roberts, Sir John and Lady Aird, the various corporation officials, Father Wyndham representing the Roman Catholic children of the schools, Dr. Adler the Chief Rabbi, representing the Jews, and the Rev. Walter Abbott. All the children had the honour of being presented to their Royal Highnesses, and then Master Jack Aird, as representative of the schools, offered for Prince Edward's acceptance an exact copy in pattern of the Coronation cup, which the King gives to his poor diners. The material however is of solid gold, with an embossed portrait of the King and Queen on one side and the young Prince's initials on the other, surrounded by a border of blue enamel, and set with precious stones, diamonds, emeralds, and rubies. On the bottom of the cup is an inscription "Presented to Prince Edward by the children of Paddington, July 1892." The beaker was enclosed in a velvet case. The ceremony was a simple one. Master Jack Aird, in sailor clothes, stepped forward, and, saluting the young prince in correct military fashion, offered the cup. Prince Edward, also clad as a sailor, gravely returned the salutation, and then heartily shook hands with the giver. The Prince of Wales returned thanks on behalf of his little son.

### Indian Princes at the India Office.

The reception given to the Indian Princes, or to give it its more correct title the "durbar," at the India Office on Friday evening last week, was a striking ceremonial. The Prince and Princess of Wales took the place of the King and Queen, and nothing more impressive has been witnessed in this country than the Oriental princes, clad in their gorgeous robes and ablaze with diamonds and precious stones, tendering their allegiance, gravely bowing to the representatives of the Emperor of India, touching their foreheads and offering the hilts of their swords to be touched by the Prince, as did the officers of the native troops. Under the crimson canopy in the magnificent hall were the Prince of Wales and the Duke of Connaught; the background was made of a semi-circle of Indians, as resplendent as only Indians can be; in front a little semi-circle formed by the Indian spearmen, and round them a huge crowd of the noblest of Englishmen and the fairest of Englishwomen. The India Office was so transformed in honour of the occasion as to be unrecognisable. The quadrangle had been covered by a huge canvas representing an Indian star-lit sky, which was illuminated by hidden lime light in such a manner as to cause the stars to twinkle and gleam as if they were real. The balustrades of the staircases were hidden by roses, ferns and palms had transformed the corridor into avenues, and ice grottoes kept the atmosphere at a pleasant temperature, whilst music from distant bands added to the charm of an entertainment quite unique in its character.

### Accident to Mr. Chamberlain.

Considerable excitement was caused on Monday afternoon by the news that Mr. Chamberlain had met with a nasty accident. The Colonial Secretary was driving in a hansom along Whitehall, and just as the vehicle passed the Canadian arch, the horse slipped and fell, the window of the hansom being shaken down. Mr. Chamberlain was thrown violently forward, and his head was jerked through the glass, with the result that a serious scalp wound was inflicted over the right temple. He was assisted from the cab and was found to be bleeding profusely. A police constable bound up his head with a handkerchief, and took him off in another hansom to Charing Cross Hospital. Here it was ascertained that the wound though not dangerous was severe, and it was thought prudent that he should remain in the hospital. He accordingly spent Monday night and Tuesday, which was his birthday, in the ward reserved for his use, where Mrs. Chamberlain and Mr. Austen Chamberlain visited him. On Wednesday Mr. Chamberlain was able to return home.

### For Reading to the Sick.

HE GIVETH REST.

God sends sometimes a stillness in our life,
The bivouac, the sleep,
When on the silent battle-field the strife
Is hushed in slumber deep,
When wearied hearts exhausted sink to rest,
Remembering nor the struggle nor the quest.

We know such hours, when the dim dewy night
Bids day's hot turmoil cease;
When star by star steals noiselessly in sight,
With silent smiles of peace;
When we lay down our load, and half forget
The morrow comes, and we must bear it yet.

We know such hours, when after days of pain,
And nights when sleep was not,
God gives us ease and peace and calm again,
Till, all the past forgot,
We say, in rest and thankfulness most deep,
E'en so "He giveth His beloved sleep."

*L. Fletcher.*

We sleep in peace in the arms of God, when we yield ourselves up to His providence, in a delightful consciousness of His tender mercies; no more restless uncertainties, no more anxious desires, no more impatience at the place we are in; for it is God who has put us there, and who holds us in His arms. Can we be unsafe where He has placed us?—*Fénelon.*

He showed a little thing, the quantity of a hazel-nut, lying in the palm of my hand, as meseemed, and it was as round as a ball. I looked thereon with the eye of my understanding, and thought, "*What may this be?*" and it was answered generally thus, "*It is all that is made.*" I marvelled how it might last; for methought it might suddenly have fallen to naught for littleness. And I was answered in my understanding, "*It lasteth, and ever shall: For God loveth it. And so hath all thing being by the Love of God.*" In this little thing I saw three properties. The first is, that God made it. The second is, that God loveth it. The third is, that God keepeth it. For this is the cause which we be not all in ease of heart and soul: for we seek here rest in this thing which is so little, where no rest is in: and we know not our God that is all Mighty, all Wise, and all Good, for he is very rest. God wills to be known, and it pleaseth Him that we rest us in Him. For all that is beneath Him, sufficeth not us.—*M. J.*, 1373.

O will, that willest good alone,
Lead Thou the way, Thou guidest best;
A silent child, I follow on,
And trusting lean upon Thy breast.
And if in gloom I see Thee not,
I lean upon Thy love unknown;
In me Thy blessed will is wrought,
If I will nothing of my own.

*Gerhard Tersteegen.*

### Notes and Queries.

**The Editor is always willing to answer in this column, without any fee, all reasonable questions, as soon as possible.**
**But the following rules must be carefully observed:—**
**1. Every communication must be accompanied by the name and address of the writer.**
**2. The question must always bear upon nursing, directly or indirectly.**
**If an answer is required by letter a fee of half-a-crown must be enclosed with the note containing the inquiry, and we cannot undertake to forward letters addressed to correspondents making inquiries. It is therefore requested that our readers will not enclose either a stamp or a stamped envelope.**

*Spring Steps.*

(117) Will you kindly tell me where I can obtain a pair of Dr. Davis's spring steps?—*P. H.*

Can you not order them through a firm that makes a speciality of nurses' requirements? There are plenty amongst the advertisers in The Hospital.

*Bugs.*

(118) Can you tell me how I can get rid of bugs? Neither "Keatings" nor carbolic have proved of any use; would you recommend fumigating the room with sulphur?—*District Nurse.*

If the room be thoroughly infested strip it and fumigate it with sulphur; then paint every crack in the woodwork, both of the wainscotting and furniture with strong carbolic acid, and inject vermite into all the cracks in the plastering, etc. Even then strict watch will have to be kept for some time for stray insects.

*Private Nursing.*

(119) Will you kindly tell me if there is any institution which I could join where I could sleep at home, and be sent out to private cases at a good salary? Would it be well for me to set up for myself in the north of London? I have a three-years' certificate for general nursing and maternity—*Nurse Ada.*

This is not a matter on which we can advise you, as so much depends on the circumstances of the individual case. There are plenty of associations which allow nurses to live at home when off duty. See our advertisement columns.

*Etiquette.*

(120) Kindly give me your opinion as to a probationer's conduct in going to a member of the visiting staff of a hospital for chest examination without the matron's knowledge. Also on the doctor's conduct in making the examination without the matron's knowledge.—*Inquirer.*

It is quite impossible for us to express any opinion on the subject.

Is it etiquette for the night superintendent to take precedence of the ward sisters in a hospital, or does she rank in accordance with her seniority?—*Puzzled.*

If the night superintendent superintends the ward sisters, of course she takes precedence of them.

*Naval Nursing Service.*

(121) Will you kindly tell me how I can get on Queen Alexandra's Nursing service?—*B. P.*

Apply for particulars of the Navy Staff of Nursing Sisters to the Director-General, Medical Department of the Navy, Admiralty, Northumberland Avenue, W.C. See also the *Nursing Section* of The Hospital for May 24, 1902.

*Suspension Treatment.*

(122) An article appeared in your paper some two or three years ago on "Suspension Treatment" for spinal disease, as carried out by Messrs. Jones and Tubby, of London, in which it was stated that the "after treatment" lasted over three years. I should be glad if you would tell me (1) what this "after treatment" consists of? (2) whether patients are still required to spend some hours daily in the suspended position, and, if so, about how long? or (3) if the "after treatment" is merely the wearing of some special form of support?—*Laminectomy.*

The treatment varies according to the case.

**Standard Nursing Manuals.**

"The Nursing Profession: How and Where to Train." 2s. net; post free 2s. 4d.
"Art of Massage." (Second Edition.) 6s.
"Elementary Physiology for Nurses." 2s.
"Elementary Anatomy and Surgery for Nurses." 2s. 6d.
"Practical Handbook of Midwifery." 6s.
"Surgical Ward Work and Nursing." Revised Edition. 3s. 6d. net; post free 3s. 10d.
"Mental Nursing." 1s.
"Art of Feeding the Invalid." 1s. 6d.
All these are published by the Scientific Press, Ltd, and may be obtained through any bookseller or direct from the publisher, 28 and 29 Southampton Street, London, W.C.

### Travel Notes.

By Our Travelling Correspondent.

### CIII.—WHAT WE CAN SEE FOR FIVE POUNDS.

### Louvain, Alost, Ghent.

Brussels is too important a place to be squeezed in with others, and must be allowed an article to itself at some future time.

Let us first go to Louvain, which is about 12 miles from Mechlin. Both times I have visited it the weather was extraordinarily hot, but we stayed in a delightful hotel, where there was a garden, and the proprietor gave us all our meals in it among his beautiful sunflowers.

The two most remarkable things in Louvain are the Hôtel de Ville and the beautiful church of St. Pierre. Motley says, with truth, that the former "is the most magnificent piece of Gothic domestic architecture in the world." It forms the background of Louis Haghe's picture, now in the South Kensington Museum, called "An Émeute in the Streets of Louvain."

There are three storeys to it, each of which has ten elaborately-decorated windows on the principal façade, and the whole thing is most lavishly enriched with sculpture. There are elegant turrets at the corners ending in graceful spires and the steep roof is enriched with numerous openings; it is very much like the town hall of Brussels, but is certainly far more beautiful. There is not much to be seen inside—nothing worth paying a fee for. Almost opposite is the charming church of St. Pierre.

### The Story of Quentin Matsys.

In this beautiful church in one of the chapels there used to be a picture of Quentin Matsys, now, alas! removed to the picture gallery at Brussels. His story is a most romantic one. He was a skilled mechanic—a kind of glorified blacksmith—and he made the beautiful well-head outside the Hôtel de Ville at Antwerp; unfortunately for his peace of mind he fell in love with one far above him in position, and was told by the girl's indignant father that never would he give his daughter to an artisan, but only to an artist.

Matsys determined to be one, and succeeded as his noble works show; it is pleasant to know that he was rewarded with the hand of the lady who had been the inspiration of so much beautiful work. There is a most perfect rood loft in this church, with three arches to it and surmounted by a cross. In the first chapel on the north side there used to be a copper font cover made by one of the Matsys family, though not Quentin, but where it has gone I could not hear.

Not far from St. Pierre is the church of Ste. Gertrude, celebrated for its magnificent wood carving. The choir stalls, made by a carver named Waydere, are said by connoisseurs to be the finest specimens of such work in Belgium.

Do not omit to visit the Weavers' Hall, now called the Halles: it was begun in 1317 and finished in 1680; inside the heavy arches on the ground floor attest its age.

### Alost.

You will see everything in Alost in a day quite comfortably. The town's greatest treasure is a picture of Rubens supposed to have been painted in a week. There is a beautiful belfry to the Hôtel de Ville, and the meat market close by is very picturesque. Alost is often despised and unvisited, but there is really much of interest there, especially to the artist; the whole effect of the Grande Place is very pleasing. I saw it very advantageously; the Kermesse was going on, and gay flags were hanging out at all the windows and lighting up the sombre buildings round the market place, whilst the gaiety of the booths and the animation of the usually stolid Flemings made the little town very bright and cheerful.

### Ghent.

This is, of course, a far more important place; it is a thriving manufacturing town; but there remains much that transports one back several centuries.

In some respects it is a more taking place than Bruges; to begin with, there is much more of it and its buildings are very grand. Ghent lives whilst Bruges dreams, but I confess that the latter holds my affections the more strongly.

The Hôtel de Ville, as always in Flanders, is grand, but in a heavier style than those we have been considering hitherto; it is very rich in ornamentation and somewhat massive, also very dark in colour. It stands in the butter market, and consists of two different parts, one built in 1518, and the other later and much more clumsy, a not very good example of the Renaissance style. Whether or no to see the interior depends, I should say, upon the state of your funds; I always grudge these 50 centime fees for seeing things which as works of art should be free to the public.

The cathedral of St. Bavon is far more interesting than one would imagine from the outside view of it, and the ancient choir stalls are charming.

The quays and squares of Ghent constitute one of its greatest attractions; the Marché du Vendredi is wonderfully picturesque, still surrounded by beautiful old houses, that have looked down on many a sanguinary brawl, such as that led by Gerard Denys, who headed weavers of the city against the fullers for some real or imaginary grievance; the battle raged so furiously that the Bishop brought out the Host to subdue the passions of the rioters, but to no purpose till upwards of 500 citizens had been slain.

The finest quay is that called Quai aux Herbes. There are on one side, close to the canal, several beautiful guild houses, especially that of the watermen. Immediately in front are moored large barges of a rich burnt sienna colour picked out with emerald green and scarlet; the whole effect is delightful. I made a sketch of the scene from the opposite side to the great entertainment of the good Flemings, but alas! the arts were interrupted by one enthusiast carrying a brazen pot of coffee, spilling it down my back, in her zeal to achieve a better view.

The belfry is not so beautiful as that of Bruges, nor are the chimes so good, but it is a pleasing object all the same. It stands in a street where there are some delightful little dim shops devoted to coppersmithing where I bought several specimens, old and new.

You must, if possible, visit the Béguinage, because, though a species of convent, the rules are quite different to those of conventual life in other countries.

This curious place forms a little town of itself to the north-east of the city. The inhabitants must be unmarried or widows and of unblemished character. Their chief work is tending the sick and needy; and they serve a novitiate of two years, after which they become sisters, though no irrevocable vows are taken.

At the expiration of six years they are allowed, if they wish to do so, to live in a small house of their own, but of course under the convent roof, if I may so express it. Their little homes somewhat resemble those of a large almshouse. Try to attend Vesper service, which is, I fancy, at 6 o'clock. The effect is very picturesque, and unlike anything one sees elsewhere.

They make a great deal of lace, and it may be bought reasonably from the Mother Superior.

### TRAVEL NOTES AND QUERIES

Accommodation in Sark (A.N.S.R.).—Write to the Hôtel Bel Air. You may use the name of Miss K. M. Bennett if you like. You do not tell me what your friends can afford, which leaves me in the dark. Tell the proprietor that the ladies mean to stay a fortnight, which always makes a difference, and ask him what are his lowest terms. If this does not suit write me again more fully, and I will tell you of places in Brittany. You might write at the same time to the Hôtel Dixcart and ask terms. At the "Victoria" they offer reasonable terms (it is a boarding house), but I have no personal experience of it.

## Figures and Tables

**Fig. 50. f1:**